# Some Medical Lessons of the War: The Control of Camp Diseases

**Published:** 1915-11-27

**Authors:** 


### SOME MEDICAL LESSONS OF THE WAR.

### The Control of Camp Diseases.

While the multiplied experiences resulting from the war will naturally include many surgical triumphs and many surgical lessons, it is none the less true that on the medical side numerous important observations are being utilised to good purpose. Whatever other results these carry, they have certainly emphasised the important part played by micro-organisms in the production of disease; the high significance of laboratory methods in the scheme of diagnosis; and the dangerous influence as spreaders of infection of "carriers" or persons in whose bodies specific germs are present though they themselves are apparently free from evil consequences. All of these conclusions have an obvious relation to the end which an Army Medical Service must necessarily cultivate above all others—namely, prevention, for here, even more than in civil life, the purpose of the profession is to keep men well and efficient. That this end has been more fully obtained during the present war than in previous records is universally allowed, and in this success necessarily the medical organisation has had a large share. While giving a full measure of credit to the influence of improved and skilfully supervised sanitation, and also to the excellent physique of the troops, due largely to a good food supply, there is overwhelming evidence to support the claims of inoculation as a protective agency against certain diseases. This has been specially conspicuous in the case of typhoid fever—the notorious scourge of armies as history plainly shows—and also in the case of tetanus and certain other complications of wounds. The hostile criticism and opposition which were active in the earlier months have for the most part subsided, and voices which speak with the authority of experience are practically unanimous in praise of the inoculation method.

### Multiple Inoculation.

Recent observations in regard to inoculation tend to confirm the absolute specificity of the several pathogenic micro-organisms responsible for the various forms of infectious disease. Thus it has been determined that, as was to be anticipated, inoculation against typhoid infection does not confer immunity to paratyphoid; and, what is not less important, that inoculation against the one infection does not increase susceptibility to the other. One issue from these and allied observations is a somewhat formidable conclusion. It is that absolute safety can only be secured by a number of inoculations, each directed to a specific end. This position is minimised by the discovery that quite satisfactory results follow the practice of simultaneous mixed inoculation. Thus Professor Dreyer reports that the injection of a vaccine containing in certain proportions typhoid bacilli, paratyphoid bacilli A, and paratyphoid bacilli B, is followed in some cases by protection against all the three infections and in other cases by protection against two of them, and this without any greater degree of immediate disturbance than is common in the ordinary antityphoid inoculation. Professor Castellani has gone even further than this, and has used what he calls a tetravaccine, which aims at protection not only against the three infections just mentioned but also against cholera. His experience in Serbia includes some 50,000 men, and he reports the entire freedom from immediate ill-effects and the practical absence from the inoculated troops of the diseases in question (*British Medical Journal*, November 13, 1915).

### Paratyphoid Fever.

The recent discussion at the Royal Society of Medicine lends prominence to the disease known as paratyphoid fever. Sir Bertrand Dawson's figures showed that of the 1,363 cases of typhoid fever reported during the war as many as 910 were proved to be paratyphoid. The variety known as paratyphoid B has been recognised in this country, but the A variety is little known here, though well defined in India. Sir W. Leishman is inclined to reject the suggestion that it has been introduced into France by soldiers who have come from India. Speaking generally, these varieties are much less severe in their clinical manifestations than the ordinary typhoid fever, and the study of them has an important bearing on civil practice. One of the plagues of the medical practitioner's life and work is the occurrence of cases which are distinguished by pyrexia and general febrile disturbance and for which it is impossible to find a cause and a confident name. Many of these are thrown into the influenza group under the necessity of finding a term which will reassure the patient. That such conditions are due to some form of bacterial invasion is a moral certainty, but the difficulty is to be sure of the specific agent. It is a fair question to raise whether among such cases there is not at least a proportion which are really examples of paratyphoid, and in dealing with individual instances this possibility ought certainly to be kept in mind. What the war has effected in this direction is to secure the opportunity for the continuous study of cases of paratyphoid fever in such number as to permit the construction of a well-defined clinical picture and to allow the working out of the conditions necessary for a certain bacteriological diagnosis. This latter is no simple matter, and the experience shows once again how important a part laboratory work must necessarily occupy in medical practice. The highly technical proceedings appear to remove such work further and further from the clinical practitioner. But it still rests with him to recognise the occasion for it and to co-operate intelligently with his laboratory colleagues. By such co-operation is to be secured the three great ends of practice—the welfare of the individual patient, the protection of the community, and the expansion of scientific knowledge. The diseases here referred to are believed to occur only occasionally and sporadically in ordinary civil practice. Possibly they would be found more frequently were the chance of their occurrence generally recognised. And in any event exemption in the past is no guarantee for the future, and especially for a future in which men who have suffered from them or have been exposed to them will return in large numbers to their British homes. In reference more particularly to the second of the two groups here suggested the risk is that some of the individuals concerned will be "carriers" of the disease and will in this fashion be sources of danger to the members of the community with whom they come in contact.

### The "Carrier" Problem.

The question of "carriers" attracted a good deal of attention in connection with outbreaks of cerebro-spinal fever which were reported some twelve months ago, and which may not improbably be repeated during the coming winter. In view of the dangers of "carriers" as sources of infection, it is obviously desirable to identify them and, if possible, to destroy the germs which they shelter. The former end may be readily attained by suitable bacteriological methods. Thus the meningococcus can be detected in the mucus collected by a swab from the naso-pharynx. Not only so, but by the influence of an abundant supply of fresh air and by the application of such agents as peroxide of hydrogen, iodine, etc., to the naso-pharynx, the "carrier" may be freed from his undesirable visitors. But even here the treatment may need some weeks before a complete result is obtained, and the problem therefore takes on an economic aspect. It may present little difficulty in the Army, where men have to do as they are told. But in civil life the position is not so easily satisfied. To compel civilians who feel in perfect health, but who happen to have been in contact, say, with a case of cerebro-spinal meningitis, to submit to medical examination, and possibly to isolation and other methods of treatment, is a formidable proposal, and a good deal of public education will be needed to effect it. There is even greater difficulty in connection with some other diseases in which the micro-organisms may be "carried." In typhoid and paratyphoid fever, for example, the recognition of the "carrier" will need an examination of the urine and fæces, and manifestly the habitat of the micro-organisms here renders efficient medical treatment much less easy of approach. So far as is known, there is no reason to believe that vaccine treatment is helpful in dealing with "carriers," and segregation, which can readily be adopted in the Army, cannot be contemplated as practicable in civil life. Hence it would seem that here we shall, as individuals and as medical advisers, have largely to rely for protection on those general measures which promote good health, and presumably, therefore, increase the capacity of the tissues to resist bacterial invasion. After all there is this comforting reflection: that the more widely spread the pathologists show us the germs of disease to be, the more certain is it that something other than the germ is necessary to successful infection. The seed and the soil, as a trite comparison has it, are both required, and if we cannot altogether prevent the scattering of the one, we can cultivate the qualities which encourage the resistance of the other. The study of micro-organisms and of "carriers," if taken alone, is well calculated to make the flesh of the public to creep; but, while not unduly minimising their dangers, there remains the broad fact that the human race continues to exist, and that with occasional and individual failures it has managed to defy successfully the onslaughts of its secret and invisible foes.

The conditions known as "traumatic neurasthenia" and "functional paralysis" were, of course, well known to physicians prior to the war, but cases of this order have been so numerous that much practical knowledge has been gained as to their management. Speculation as to the nature of such cases on their psychical side can hardly be said to have been advanced by these experiences, but the need for wise treatment, including both mental and moral elements, has been reinforced. A point of much practical importance, emphasised by Dr. Wilfred Harris at the recent discussion at the Medical Society on gunshot injuries of the nerves, is the necessity of recognising that a paralysis following an injury may in its distribution be partly organic, that is, due to the nerve injury, and partly hysterical or functional. Unless this is recognised there is danger in individual cases of coming to a very inaccurate diagnosis relative to the site of the lesion, and this is the more important seeing that the diagnosis here is very likely to be followed by surgical interference. There is a sound medical doctrine that because a patient has some functional or hysterical symptoms it by no means follows that she has not also organic disease, and this is taught more particularly in the study of disseminate sclerosis.

### An Ingenious Idea.

It may sound strange to include among the medical lessons of the war the dressing of wounds, but among the suggestions advanced by Sir Almroth Wright is one to the effect that there shall be instituted a "physician in charge of wounds." The well-worn lines about the wise physician skilled our wounds to heal have always been somewhat of a surgical grievance when quoted by the journalist or the after-dinner speaker. It is the wound infection which is the serious element in the wound, and, so far as this is concerned, the antiseptic system, according to Sir Almroth Wright, has completely broken down. The technique of the methods he proposes to substitute needs a very special training, and hence, while leaving operative measures to the surgeon, he would place in each hospital unit an officer having clinical and laboratory experience who would exercise supervision as "a physician in charge of wounds" over all the departments of treatment which lie outside the sphere of operative surgery.

